# Imperforate anus with rectopenile fistula: a case report and systematic review of the literature

**DOI:** 10.1186/s12887-016-0604-z

**Published:** 2016-05-13

**Authors:** Gang Yang, Yingli Wang, Xiaoping Jiang

**Affiliations:** Department of Pediatric Surgery, West China Hospital, Sichuan University, Chengdu, 610041 People’s Republic of China; Department of Hematology, West China Hospital, Sichuan University, Chengdu, 610041 People’s Republic of China

**Keywords:** Anorectal malformation, Rectopenile fistula, Systematic review

## Abstract

**Background:**

Although anorectal malformations (ARMs) are frequently encountered, rare variants difficult to classify have been reported.

**Methods:**

This study describes a patient with ARM and rectopenile fistula. The literature was reviewed systematically to assess the anatomical characteristics, clinical presentations and operations of this rare type of ARM.

**Results:**

Eight patients were reported in the six included articles. In three patients, the fistula extended from the rectum to the anterior urethra without communication with the skin. In one patient, the fistula, located deep in corpus spongiosum, opened to the ventral aspect of the penis without communication with the urethra. In the remaining four patients, the fistula extended from the rectum to the cutaneous orifice in the ventral aspect of penis, with communication or a short common channel with the urethra.

**Conclusions:**

Imperforate anus with fistula extending into the penis is a rare variant of anorectal malformation. Unawareness of this lesion resulted in a delay of correct diagnosis and appropriate management. A thorough examination, including colonourethrography and fistulography, should be performed in all patients with a fistula opening in the ventral aspect of the penis.

## Background

Anorectal malformations (ARMs) are frequently encountered anomalies of diverse types. Most types of ARMs can be determined by a thorough perineal inspection or colostogram. Some rare variants, however, may be difficult to classify. This report describes a rare form of ARM with a fistula opening in the ventral aspect of the penis and communicating with the urethra. The literature was reviewed systematically to assess the anatomical characteristics, clinical presentations and operations of this rare type of ARM. Understanding of this lesion is critical for early diagnosis and appropriate treatment.

## Case report

**Fig. 1:**
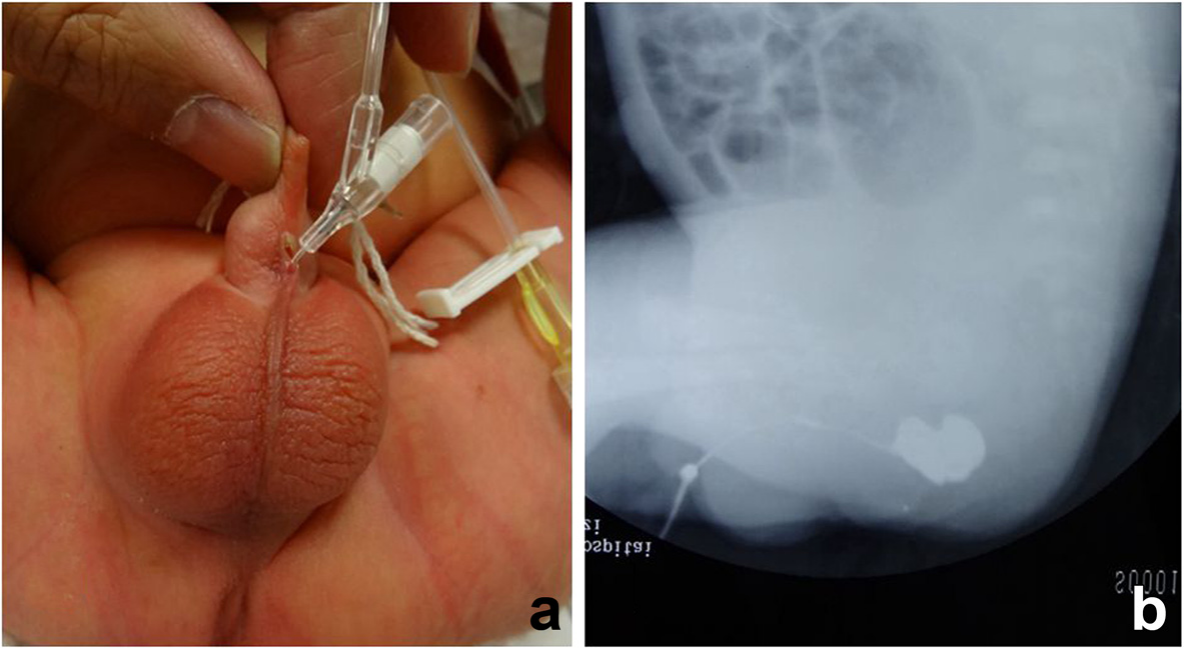
**a** A catheter was inserted into the orifice of the fistula. **b** Fistulography showed the fistula and the end of the rectum

**Fig. 2:**
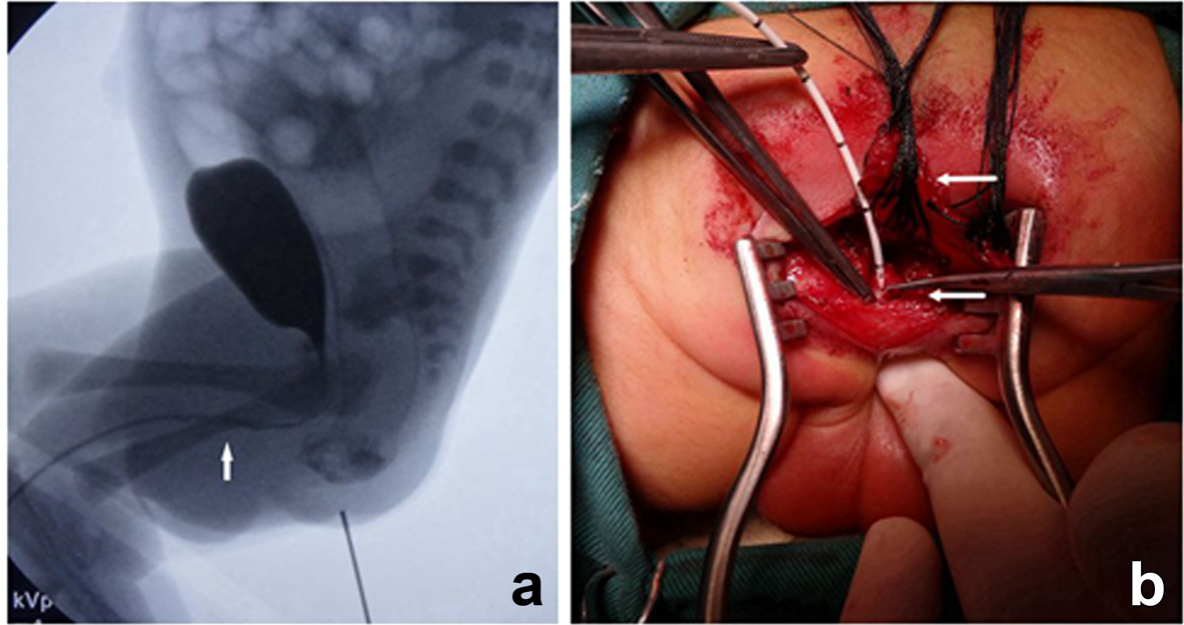
**a** Urethrography showed the urethra and the bladder. A small amount of contrast appeared in the rectum. The location of fistula entering the urethra was displayed (arrow). **b** Limited PSARP was performed. The rectum had been divided (upper arrow) and a catheter was inserted to the fistula (lower arrow)

A 4-h-old male newborn weighing 3,400 g was referred to our hospital with an imperforate anus. There was no orifice in the perineal region. A white median raphe cyst, 4 mm in diameter, was present on the ventral side of the penis. Auscultation of the heart and lungs was normal. The patient’s family history was unremarkable. After 24 h, the color of the middle raphe cyst turned dark green. The cyst was incised at bedside, and meconium passed from it. Insertion of a soft catheter showed a deep fistula running parallel to the urethra (Fig. 1a). Urination was normal, with no urine passed from the fistula. Urethrography and fistulography, performed 3 and 4 days after birth, respectively, showed a long fistula running parallel to the urethra from the rectal pouch to the penis (Fig. 1b). The distance between the rectal pouch and the anal dimple was 1 cm. During urethrography, a small amount of contrast retrograde had flowed into the rectal pouch (Fig. 2a), suggesting a communication between the fistula and the urethra. X-rays and ultrasound did not show any anomalies in the sacrum and spinal cord. No other anomalies could be identified.

One-stage limited posterior sagittal anorectoplasty (PSARP) was performed on the fourth day after birth. With the urethral catheter indwelling, the patient was placed in the prone position, and a sagittal incision was made. The posterior rectal wall was opened in the midline and extended distally, ending directly at the fistula, which was 2 mm in diameter. The dissection continued between the rectum and the urethra until the two structures were completely separated from each other. The distal end of the fistula was ligated to the remaining part of the corpus spongiosum penis (Fig. 2b). The muscles were repaired and anorectoplasty was performed. The urethral catheter was removed on the seventh postoperative day and there was no difficulty in urination. Patient recovery was uneventful and he was discharged on the eighth postoperative day. Follow-up for eight months has shown no evidence of secretion passing from the opening of penile fistula, and the patient has been doing well. Bowel control could not be evaluated owing to patient age.

## Methods

The systematic review of patients with imperforated anus and rectopenile fistula adhered to PRISMA guidelines. PubMed and EMBASE (from January 1989) were systematically searched for relevant articles published in English through November, 2014, using as search terms: (anorectal malformation OR imperforate anus) AND (penis fistula). The titles and abstracts of all potential relevant articles were read to determine their relevance. Full articles were also scrutinized if the title and abstract were unclear. Reference lists of identified articles were screened for additional publications of interest.

Studies were included if they assessed patients with ARM having fistulas extending from the rectal pouch to the penis deep in the spongy urethra, with or without connections to the urethra. Studies were also included if they described treatment details and showed radiographic images. Only studies published in English were included. Review articles and editorials were excluded.

All identified articles were independently assessed by two authors. Detailed data regarding study design, patient characteristics, initial diagnoses, radiological diagnoses, symptoms, and treatment were extracted into an electronic data sheet in a standardized manner.

## Results

**Fig. 3:**
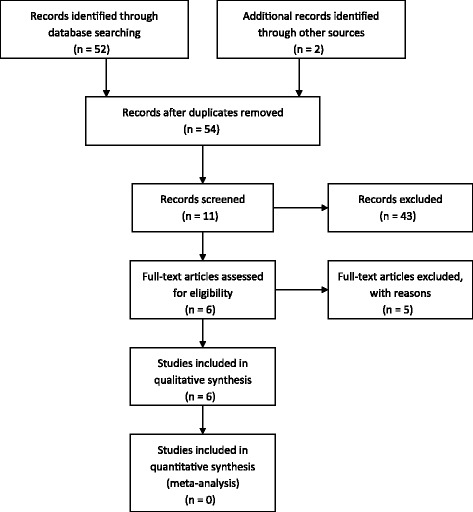
PRISMA flow chart of literature search

Of the 52 papers identified by searching the databases, four met the study criteria and were included [1–4]. No reports were repetitive. Two additional articles were identified by manual searching [5, 6]. The 48 papers excluded were review articles, irrelevant to the current study or published in a language other than English (Fig. 3). Table 1 shows the details of the included articles.

**Table 1:** Summary of included cases

Authors	Year	Age of Diagnosis	Type of Fistula	Level of rectum (distance to skin)	Operation	Management of fistula	Associated anomalies	Complications
Ohno et al.	2008	6 months	parallel to the urethra from the rectal pouch to the spongy urethra	Below the ischium (1 cm)	Transverse colostomy, ASARP, colostomy clousure	Severed from the rectum and ligated	Right aortic arch	Vesicoureteral reflux, constipation
Kumar et al.	2005	18 months	between the anal canal and the skin in the peno-scrotal junction, with a small portion of common channel in the penile urethra		Anoplasty, colostomy + fistula excision	removed		
Shah et al.	2003	9 months	From the rectum to the ventral aspect of the penis, no communication with urethra	low	Transverse colostomy, PSARP, colostomy closure	Ligated, distal part was kept undisturbed	Solitary kidney	
Currarino et al.	1994	9 months	extending from rectum to cutaneous orifice near the penoscrotal junction, with communication with the bulbar urethra	Below the ischial line	Perineal anoplasty, descending colostomy, fistula excision		Bifid scrotum, mild sacral anomalies	Urinary tract infection
		2 days	A long rectocutaneous fistula open on the undersurface of the penis, communicating with the bulbar urethra	Below the ischial line	Colostomy, sacroperineal rectal pull-through with ligation of rectal fistula, colostomy closure, excision of urethrocutaneous fistula		Bifid scrotum	
Takamatsu et al.	1993	11 months	Fistula between the anorectum and anterior urethra	below the I line	Sigmoid colostomy, perineal anoplasty and revision of the fistula		Bifida scrotum, hypospadias, right undescended testicle, right hydronephrosis, congenital heart disease	
		Unknown	Fistula between the anorectum and anterior urethra	Below the I line	Sigmoid colostomy, revision of fistula and perineal anoplasty			
Asano et al.	1983	3 months	Fistula from rectum and open in the ventral surface of the penis, communication with urethra	Under the skin	Cutback procedure, excision of the fistula			

ASARP: anterior sagittal anorectoplasty; PSARP: posterior sagittal anorectoplasty

The six included articles described a total of eight patients. In all eight, the ends of the rectum were below the ischial line. In three patients, the fistulas extended from the rectum to the anterior urethra without communicating with the skin. In one patient, the fistula, located deep in the corpus spongiosum, opened to the ventral aspect of the penis without communicating with the urethra. In the other four patients, the fistulas extended from the rectum to the cutaneous orifices on the ventral aspect of the penis, communicating with or sharing a short common channel with the urethra. Each of the eight patients underwent two to four operations. Although having a low type of ARM, seven patients underwent colostomy owing to the puzzling courses of the fistulas. Anoplasty was completed by perineal operations in six patients, an anterior sagittal approach in one and a posterior sagittal approach in one. In two patients, the fistulas were not removed because they did not communicate with the urethra. Five of these eight patients had other anomalies, including congenital heart disease, bifid scrotum, solitary kidney, hypospadias, undescended testis and hydronephrosis.

## Discussion

**Fig. 4:**
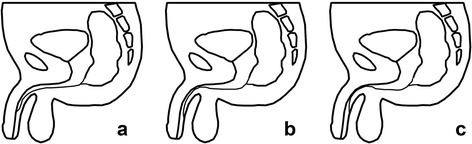
Diagrams for the different type of malformations (**a** anopenile urethral fistula; **b** fistula extending in the corpus spongiosum and opening in the ventral aspect of the penis; **c** Fistula with a communication or a short common channel with the urethra)

ARM with fistula deep in the spongy urethra, with or without communication with the urethra, is a rare anomaly [7]. The nine patients reported to date can be classified into three groups (Fig. 4). The first group consists of patients with an imperforate anus and a fistula running parallel to the urethra, extending from the rectal pouch to the anterior urethra, a condition defined as anopenile urethral fistula. The second group consists of patients with an imperforate anus and a fistula extending to the corpus spongiosum and opening in the ventral aspect of the penis. The third group consists of patients with an imperforate anus and a fistula passing distally within the corpus spongiosum and ending in the ventral aspect of the penis, with a communication or a short common channel with the urethra.

The embryology of these anomalies has not been determined. One study reported that this condition was a variant of anorectal malformation with a deep recto-cutaneous fistula that may partially fuse with the urethra [6]. The patient described in this report could be classified into the third group. A filiform fistula must have been present between the rectal pouch and the urethra, because the contrast retrograde flowed into the rectal pouch during urethrography.

Determination of anatomy is important in the management of ARM. Thorough perineal inspection may provide important clues about the anatomical type [8]. Median raphe cysts usually occur in low-type anorectal malformations and suggest the location of the fistula [9]. In our patient, a probe tube was inserted into the fistula after incision of the median raphe cysts. A fistula was observed deep within the urethra cavernosum, excluding the possibility of a perineal fistula. Urethrography and fistulography with water soluble contrast radiography were important in delineating the fistula in all eight patients.

Anorectoplasty in patients with “low type” malformation can be completed through a perineal, anterior sagittal or limited posterior sagittal approach [10]. Bowel control was excellent in most reported cases. Diverting colostomy may be unnecessary, providing that the anatomical structure is understood and delineated before surgery. Colostomy, however, is prudent if there is any suspicion about the anatomy. Fistula management should be individualized, with no uniform recommendations made. The origin of the fistula from the rectum must be ligated. The distal part of the fistula may be kept undisturbed or removed based on its relationship with the urethra, the estimated risk of infection and the difficulty of the procedure.

## Conclusions

Imperforate anus with the fistula extending into the corpus spongiosum is rare, but good prognosis can be achieved by appropriate treatment. However, lack of awareness of these lesions may delay a correct diagnosis, putting patients at risk of multiple operations. Therefore, patients with a fistula opening in the ventral aspect of the penis should be thoroughly examined, including by colonourethrography and fistulography, to clarify their anatomy before surgery.

### Consent

Written informed consent was obtained from the patient’s parents for publication of this Case report and any accompanying images. A copy of the written consent is available for review by the Editor-in-Chief of this journal.

### Ethical statement

This study was approved by the Ethics Committee of West China Hospital.

### Availability of data and materials

Not applicable.
